# Merging and comparing three mitochondrial markers for phylogenetic studies of Eurasian reindeer (*Rangifer tarandus*)

**DOI:** 10.1002/ece3.2199

**Published:** 2016-06-01

**Authors:** Kjersti S. Kvie, Jan Heggenes, Knut H. Røed

**Affiliations:** ^1^ Department of Environmental StudiesUniversity College of Southeast NorwayBø in TelemarkNorway; ^2^ Department of Basic Sciences and Aquatic MedicineNorwegian University of Life SciencesOsloNorway

**Keywords:** COI, CR, cytb, mitochondrial markers, phylogeny, *Rangifer tarandus*

## Abstract

Phylogenetic analyses provide information that can be useful in the conservation of genetic variation by identifying intraspecific genetic structure. Reconstruction of phylogenetic relationships requires the use of markers with the appropriate amount of variation relative to the timeframe and purpose of the study. Here, genetic structure and clustering are inferred from comparative analyses of three widely used mitochondrial markers, the CR, cytb and the COI region, merged and separately, using Eurasian reindeer as a model. A Bayesian phylogeny and a MJ network, both based on the merged dataset, indicate several distinct maternal haplotype clusters within Eurasian reindeer. In addition to confirm previously described clusters, two new subclusters were found. When comparing the results from the merged dataset with the results from analyses of the three markers separately, similar clustering was found in the CR and COI phylogenies, whereas the cytb region showed poor resolution. Phylogenetic analyses of the merged dataset and the CR revealed congruent results, implying that single sequencing analysis of the CR is an applicable method for studying the haplotype structure in Eurasian reindeer.

## Introduction

Genetic diversity is the raw material for evolution which allows species to adapt to environmental change and to evolve local adaptations (Conner and Hartl 2004). Identification of intraspecific genetic structure and variation through phylogenetic analyses is therefore an important area in species conservation (Avise 2000). A wide range of molecular markers are presently available, and the loci of choice for most phylogenetic studies are located on the mitochondrial genome (Gissi et al. 2008). Mitochondrial DNA (mtDNA) is a popular genetic marker due to qualities such as high mutation rate, maternal inheritance and high prevalence in the cells, making it easy to amplify (Avise 2000; Gissi et al. 2008). MtDNA show high intragenomic variability, and substitution rates depend on which region that is considered (Pesole et al. 1999). Variation within the regions is also found; for example, the mitochondrial control region (CR), also called the displacement loop (D‐loop), consists of selectively neutral blocks showing high mutation rates in addition to a more conserved central domain (Reyes et al. 2003). The highly variable regions are frequently used for intraspecific studies, whereas the conserved region is used for studies of more diverged taxa (Reyes et al. 2003). Other commonly used mitochondrial markers are the protein‐coding cytochrome b (cytb) and cytochrome *c* oxidase subunit 1 (COI) regions. These markers are often used for examining deeper splits within species (Kurose et al. 1999; Ursenbacher et al. 2006; Kvie et al. 2013), in addition to being popular markers in species delimitation and in phylogenetic studies above species level (Hebert et al. 2003; Tobe et al. 2010). A potential problem associated with markers showing elevated substitution rates is the loss of information and underestimation of relationships between populations due to homoplasy, that is, sequence similarities due to convergent, parallel, or reverse evolution rather than common ancestry (Ballard and Rand 2005). Using additional, protein‐coding markers that are more conserved as control might be a solution to avoid potential bias due to homoplasy. However, a shortcoming of using protein‐coding mtDNA as a phylogenetic marker is possible selection owing to metabolic requirements (Foote et al. 2011). Although assumed to evolve in a neutral manner, recent studies have questioned this assumption and selection on mtDNA regions has been detected in several species (Castoe et al. 2008; da Fonseca et al. 2008; Foote et al. 2011).

**Figure 1:**
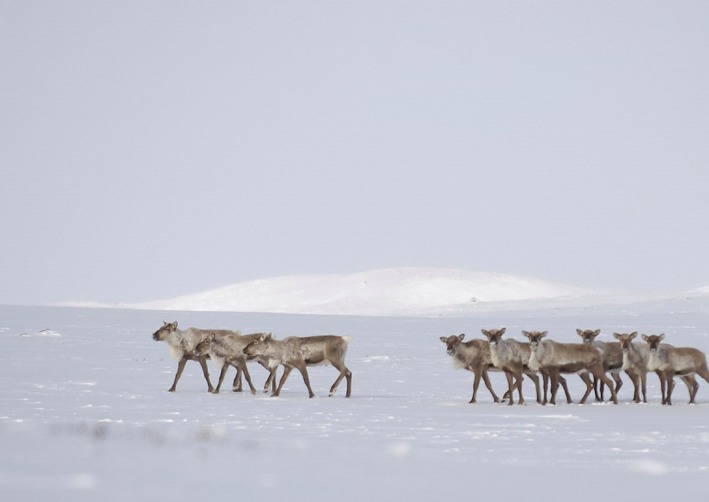
Wild tundra reindeer (*Rangifer tarandus tarandus*) on Hardangervidda, Norway. Photograph: Arvid Haga.

Reindeer (*Rangifer tarandus*) (Fig. 1), a migratory ungulate in the family Cervidae, have been widely distributed in most mountain, tundra and taiga areas throughout the northern Holarctic during most of the Holocene. Reindeer have been classified into three ecological groups: woodland, tundra and high arctic island forms, based on morphological and historical data (Banfield 1961). These ecological groups include nine subspecies, of which seven are extant. However, previous genetic studies based on mitochondrial DNA indicate discrepancies between current ecotype and subspecies designations and genetic differentiation (Gravlund et al. 1998; Flagstad and Røed 2003; Cronin et al. 2006; McDevitt et al. 2009). This may imply that the current geographical distribution of mtDNA haplotypes reflects historical demographic events rather than taxonomy as it is described today. Previous studies on reindeer haplotype structure are mainly based on single sequencing data from either the CR (Flagstad and Røed 2003; Røed et al. 2008; Kholodova et al. 2011; Klütsch et al. 2012; Weckworth et al. 2012) or the cytb region (Cronin et al. 2006; Yannic et al. 2014), but we are not aware of any combined studies. Phylogeographical analyses of reindeer based on the CR show three main haplotype clusters, probably originating in three separate glacial populations. The largest cluster comprises reindeer from Eurasia as well as North America, pointing toward a Beringian origin. The North American and sedentary woodland caribou (*R.t. caribou*) constitutes a second main CR cluster with haplotypes probably originating south of the Wisconsinan ice sheet (Flagstad and Røed 2003). Finally, a third cluster comprising haplotypes with a purely Eurasian origin is suggested to have undergone recent isolation, probably in connection with ice expansion in Eurasia during the Weichselian (Flagstad and Røed 2003). Two of the three clusters above have in later studies been further subdivided. The Beringian cluster shows six CR subclusters within Eurasian reindeer (Røed et al. 2008; Bjørnstad et al. 2012), while three lines within the North American woodland caribou have been described (Klütsch et al. 2012). The Beringian cluster and the North American cluster have also been identified through the phylogenetic reconstruction of the cytb region (Yannic et al. 2014).

Reindeer, as well as other Holarctic species, may be threatened by climate change and other anthropological impacts, and therefore, clear identification of phylogenetic structure below the species level is important in order to protect genetic variation (Vogler and Desalle 1994; Bloor et al. 2015). Here, we test the phylogenetic performance of three mitochondrial markers, merged and separately, using Eurasian reindeer as a model species. In addition to the much used CR and cytb region, a fragment from the COI region is included to obtain a longer fragment from the coding part of the mitochondrial genome. First, we want to merge the sequence data from the CR, cytb and the COI region to take advantage of the three markers’ ability to resolve relationships on different taxonomic levels, as this may lead to increased resolution and clearer delineation of phylogenetic groups (Knaus et al. 2011). Second, we want to see how the three markers perform individually by comparing the variability found in each marker. It would be reasonable to expect these markers to show compatible results, as the mitochondrial genome can be considered a single locus and hence the three markers are principally linked (Avise 2000). However, properties of neutral variation in the CR compared to the functional gene parts of the mtDNA genome may result in different phylogenetic patterns due to different levels of resolution (Avise 2000).

## Material and Methods

### Study material

DNA from a total of 183 skin, muscle and blood samples were extracted and analyzed. The skin samples were stored dry and cold, the muscle samples in ≥ 80% ethanol, and the blood samples in EDTA. The samples were collected in Norway/Svalbard (*n* = 137) and Russia (*n* = 46), covering the northwestern parts of Eurasia (Table 2). A major part of the samples were collected in Norway to include the variation and structure found in the margins of the species distribution area. The sample set comprises wild tundra reindeer from south‐central parts of Norway (Hardangervidda and Rondane/Dovre), reindeer from the Svalbard archipelago, representing the wild high arctic type, and reindeer from northern Norway (*n* = 27), representing the domestic Scandinavian tundra type. Wild reindeer from western Taimyr as well as domesticated reindeer from Kola/Yamal were included representing Russian tundra reindeer.

### DNA extraction and amplification

DNA was extracted using DNAeasy Blood & Tissue Kit (Qiagen, Inc., Valencia, CA) following the manufacturer's protocol. A 503‐base pair‐long fragment from the mitochondrial control region was amplified using the forward primer RtCRF‐(0‐) (5′‐AAT AGC CCC ACT ATG AGC ACC‐3′) (Flagstad and Røed 2003) and the reverse primer RtCR‐(528) (5′‐TAG GTG AGA TGG CCC TGA AGA AA‐3′) (Bjørnstad and Røed 2010). Amplification was performed using the following program: 95°C for 2 min, 95°C for 30 sec, 55°C for 30 sec, and 72°C for 1 min (steps 2–4 cycled 30 times) and finally 72°C for 10 min. Amplification of a 308‐bp‐long fragment from the cytb region was performed using the primers cytb F (5′‐CCATCCAACATCTCAGCATGATGAAA‐3′) and cytb R (5′‐GCCCCTCAGAATGATATTTGTCCTCA‐3′). The PCR program for this primer pair was as follows: 95°C for 5 min, 95°C for 45 sec, 53–55°C for 40 sec, and 72°C for 2 min (steps 2–4 cycled 32 times) and finally 72°C for 10 min. For amplification of a 657‐bp‐long COI fragment, minor modifications were made to a primer pair originally used for antelopes, COIbF/COIbR (Bitanyi et al. 2011), using the primer design program Primer3Plus (Untergasser et al. 2007). Amplification was performed with the primer pair Rt_COIF (5′‐TCACAAAGACATTGGCACCT‐3′) and Rt_COIR (5′‐TGATTCTTTGGACACCCTGA‐3′). The PCR profile used was as follows: 94°C for 1 min, 94°C for 30 sec, 50°C for 40 sec, 72°C for 1 min (steps 2–4 cycled 5 times), 94°C for 30 sec, 54°C for 40 sec, 72°C for 1 min (steps 5–7 cycled 35 times), and 72°C for 10 min. PCRs were performed using reaction mixture containing 1–2 *μ*L DNA template, 1X buffer, 1.5 mmol/L MgCl_2_, 0.8 mmol/L dNTPs, 5 pmol of each primer, 0.5 *μ*g/*μ*L bovine serum albumin (BSA), 0.5 U/*μ*L AmpliTaq DNA polymerase (Applied Biosystems, Foster City, CA) and dH_2_O to make up the total volume of 20 *μ*L.

### Sequencing

The samples were cleaned for unincorporated primers and nucleotides using Illustra ExoProStar (GE Healthcare, Buckinghamshire, UK) diluted 10 times. Cycle sequencing was performed in a 10‐*μ*L reaction volume, using BigDye v3.1 sequencing kit (Applied Biosystems) following the manufacturer's recommendations. Purification was carried out using standard EDTA/EtOH precipitation. Capillary electrophoresis and data analysis were performed with an ABI 3130xL or 3500xL instrument (Applied Biosystems). All sequences were sequenced in both directions, and the consensus sequences were aligned by ClustalW (Thompson et al. 1994) and edited in MEGA v.5.2 (Tamura et al. 2011). We used Mega v.5.2 to manually concatenate the sequenced fragments from the three regions (CR, cytb, and COI).

Using highly conserved primers increases the chance of amplifying nonfunctional copies of mtDNA (numts), which might be a source for erroneous interpretation of the data (Gelissen et al. 1983; Galtier et al. 2009). Alignments produced from the coding parts of the mitochondrial genome (cytb and COI) were controlled for the presence of numts by translating the alignments from nucleotide to amino acid sequences to check for stop codons and frameshift mutations.

### Statistical analyses

DNA polymorphism calculations (the number of haplotypes, gene diversity, and nucleotide diversity) were performed in DnaSP v5.10 (Librado and Rozas 2009) for each locus and for the different geographical regions**.** DnaSP was also used to test for neutrality by calculating Tajima's *D* (Tajima 1989) as selectively neutral markers will give more precise estimates of genetic variation and structure, compared to a marker under selection (Avise 2000). A negative Tajima's *D* corresponds to an excess of rare polymorphisms and implies a population size expansion or positive selection, while the positive values reflect an excess of intermediate‐frequency alleles, and suggests population bottlenecks or balancing selection (Akey et al. 2004). All four alignments were reduced to include only one sequence of each haplotype for the phylogenetic analyses. We used BEAST v1.8.0 (Drummond et al. 2012) to construct Bayesian phylogenies for the three regions combined and separately. For the combined analyses, a single gene tree was constructed as all three markers are mitochondrial and therefore linked. We partitioned the datasets into three regions (cytb, COI and CR) to allow for different substitution models and rates for each locus. We used PartitionFinder v.1.1 (Lanfear et al. 2012) to identify the optimum partition scheme and substitution models for the two coding regions. The cytb region was partitioned into 1st, 2nd, 3rd codon position, while the COI region was partitioned into two partitions, (1st + 2nd) and 3rd. We used the HKY and the HKY + I + G substitution model for the cytb/COI regions and the CR, respectively. The substitution rate was set to 2.23%/Myr for the cytb region (Yannic et al. 2014) and 58.9%/Myr for the CR (Ho et al. 2008). Based on the settings above, the substitution rate for the COI region was estimated in BEAST and subsequently set to 7.6%/Myr. Sequences (*n* = 11) from previously described CR haplotype clusters were included in the CR Bayesian analysis to designate sequences from the current study to previously described haplotype clusters (Røed et al. 2008; Bjørnstad et al. 2012). The analyses were run for 100 000 000 generations and 10% of the initial samples removed as burn‐in. Convergence for the phylogenies generated in BEAST was assessed in TRACER (Rambaut et al. 2014), and the effective sample size for all parameters was above the general recommendation (ESS > 200). We also used BEAST v1.8.0 to calculate time since most recent common ancestor (tMRCA) to test whether the different CR haplotype clusters/subclusters diverged during or after the last glacial maximum (LGM). The molecular clock, model of substitution, and the number of generations were set as described above for the CR. The stem option was not implemented so that the time reported reflects tMRCA of the taxon set (the haplotype cluster). A median‐joining network (Bandelt et al. 1999) based on the full, concatenated dataset (*n* = 183) was constructed using Network v4.6 (ref.fluxus‐engineering.com). A MJ network should give an appropriate presentation of the intraspecific variation found, as it, unlike bifurcating phylogenetic methods, takes into account the fact that intraspecific relationship tends to include extant ancestral haplotypes and multifurcations (Posada and Crandall 2001).

## Results

### Sequence data and genetic variation

A total of 1284 bp from 183 individuals were analyzed for the CR, cytb and the COI region (GenBank accession numbers CR: KX094568‐KX094750, cytb: KX066893‐KX067075, and COI: KX085230‐KX085412). To include as many sequences as possible, the CR alignment was trimmed from 503 or 504 bp (depending on the presence of a thymine indel) to 467 or 468 bp. The cytb dataset was analyzed in its full length of 308 bp, while the COI dataset was trimmed from 657 bp to 508 bp. Translating sequences from the two coding mtDNA regions revealed no stop codons or frameshift mutations in the alignments. All substitutions were synonymous except from one individual, with a single nonsynonymous substitution in the cytb region.

Standard estimates of DNA polymorphism in the merged dataset showed a high degree of variation with 54 haplotypes and haplotype (hd) and nucleotide (*π*) diversity equal to 0.917 and 0.010, respectively (Table 1). The CR dataset, analyzed separately, also showed a high degree of haplotype and nucleotide diversity (hd = 0.908, *π *= 0.020) and comprised 46 haplotypes (Table 1). In the cytb fragment, 13 haplotypes were found (hd = 0.731 and *π *= 0.005), whereas the COI region identified 18 haplotypes (hd = 0.856 and a *π *= 0.004) (Table 1). None of the datasets deviate from a neutral model with a constant population size; that is, none of the Tajima's *D* estimates are significantly different from zero (Table 1). Standard estimates of DNA polymorphism for each locus show large differences in variability in the geographical regions under study (Table 2). Haplotype diversity and nucleotide diversity are lowest within the wild populations from Svalbard/Norway (Svalbard and Rondane/Dovre), while the highest levels of DNA polymorphisms are found in wild reindeer from Russia (Taimyr). The wild population from Hardangervidda and the domestic populations from Norway (Finnmark) and Russia (Kola/Yamal) show variation within a more intermediate range (Table 2).

**Table 1:** Tajima's *D* and genetic variability in Eurasian reindeer (*n* = 183) based on the three mtDNA markers analyzed individually and merged

	*N* a	*L* b	Tajima's *D* c	*H* d	Hd^e^	*π* f
CR	183	468	0.170	46	0.908	0.020
cytb	183	308	−0.860	13	0.731	0.005
COI	183	508	−1.011	18	0.856	0.004
Merged	183	1284	−0.344	54	0.917	0.010

a The number of individuals.

b Sequence length.

c Neutrality test to measure whether the data deviate from the expectations under a neutral model with the constant population size. None of the Tajima's *D* values are significant (*P *>* *0.10).

d The Number of haplotypes.

e Haplotype (gene) diversity.

f Nucleotide diversity.

**Table 2:** DNA polymorphism in the CR, cytb, and the COI region for the different geographical regions

Area	*N* a	CR			cytb			COI			Merged		
		*H* b	Hd^c^	*π* d	*H* b	Hd^c^	*π* d	*H* b	Hd^c^	*π* d	*H* b	Hd^c^	*π* d
Hardangervidda	30	10	0.811	0.017	5	0.639	0.004	5	0.644	0.003	11	0.818	0.008
Rondane/Dovre	53	4	0.437	0.008	2	0.142	0.001	3	0.380	0.001	4	0.437	0.004
Svalbard	27	3	0.501	0.001	1	0.000	0.000	2	0.205	0.000	4	0.630	0.001
Finnmark	27	8	0.695	0.014	4	0.533	0.003	4	0.573	0.003	11	0.798	0.007
Taimyr	26	20	0.972	0.017	10	0.843	0.006	10	0.898	0.006	20	0.972	0.009
Kola/Yamal	20	10	0.758	0.015	5	0.616	0.004	8	0.742	0.005	11	0.842	0.009

a Sample size.

b Number of haplotypes.

c Haplotype (gene) diversity.

d Nucleotide diversity.

### Haplotype structure based on the merged dataset

**Figure 2:**
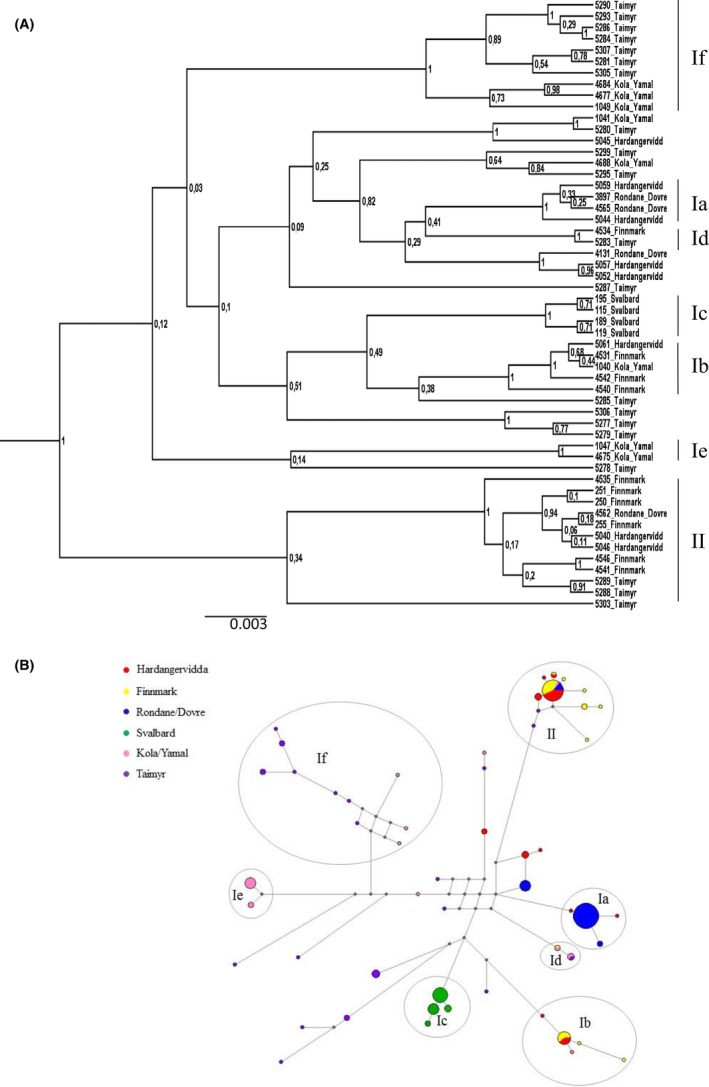
(A) Bayesian phylogeny of the three datasets combined, showing 54 haplotypes and well‐supported subclusters **Ia–If** and cluster **II** (posterior probability values shown at each node). We found a main division between clusters **I** and **II** as well as six subclusters within cluster **I**. (B) Median‐joining network of the concatenated dataset (1284 bp) from the CR, cytb, and COI region (*n* = 183) showing subclusters **Ia–If** and cluster **II** **.** The size of the circles corresponds to the number of individuals with the same haplotype. Pink: domesticated reindeer from Yamal/Kola, Russia. Purple: Wild reindeer from Taimyr, Russia. Green: Wild reindeer from the Svalbard archipelago, Norway. Yellow: Domesticated reindeer from Finnmark, northern Norway. Blue: Wild reindeer from Rondane/Dovre, central parts of Norway. Red: Wild reindeer from Hardangervidda, southern parts of Norway. The gray circles show the clusters/subclusters that obtained high support in the Bayesian phylogeny based on the three regions combined (A).

The Bayesian phylogeny and the MJ network indicated a high degree of variation, and several well‐supported clusters/subclusters were identified (Fig. 2A and B), some of which have been described in previous studies based on the CR (Røed et al. 2008; Bjørnstad et al. 2012). In the current study, new subclusters were defined when showing high support (≥90), comprising ≥4 haplotypes in addition to showing some degree of geographical structure. Clusters that have been described in previous studies were included when showing ≥2 haplotypes. The merged dataset shows a main division between clusters **I** and **II,** with six subclusters identified within cluster **I**. Subcluster **Ia** includes 4 haplotypes (*n* = 43) found in wild reindeer from Rondane/Dovre, except for two samples from Hardangervidda. Subcluster **Ib** comprises five haplotypes (*n* = 14) found in both wild and domestic reindeer from Hardangervidda, Finnmark, and Kola/Yamal. Haplotype cluster **Ic** includes 4 haplotypes (*n* = 27) that are all unique for Svalbard reindeer. Two smaller subclusters denoted **Id** and **Ie**, both comprising two haplotypes, were also identified. **Id** haplotypes (*n* = 5) were found in domesticated reindeer from Kola/Yamal and Finnmark, and in wild reindeer from Taimyr, whereas haplotypes in subcluster **Ie** (*n* = 10) were found in domesticated reindeer from Kola/Yamal. The highly variable subcluster **If** comprises 10 haplotypes (*n* = 12) found in wild reindeer from Taimyr and in domesticated reindeer from Kola/Yamal. Finally, cluster **II** includes the most diverged haplotypes and is separated from other clusters with a minimum of 11 mutations in the MJ network (Fig. 2B, mutations not shown). This cluster includes 11 haplotypes (*n* = 42) found mainly in domesticated reindeer from Finnmark, in addition to samples from Kola/Yamal, Hardangervidda, Rondane/Dovre, and the Taimyr area.

### Haplotype structure based on the three markers analyzed separately

**Figure 3:**
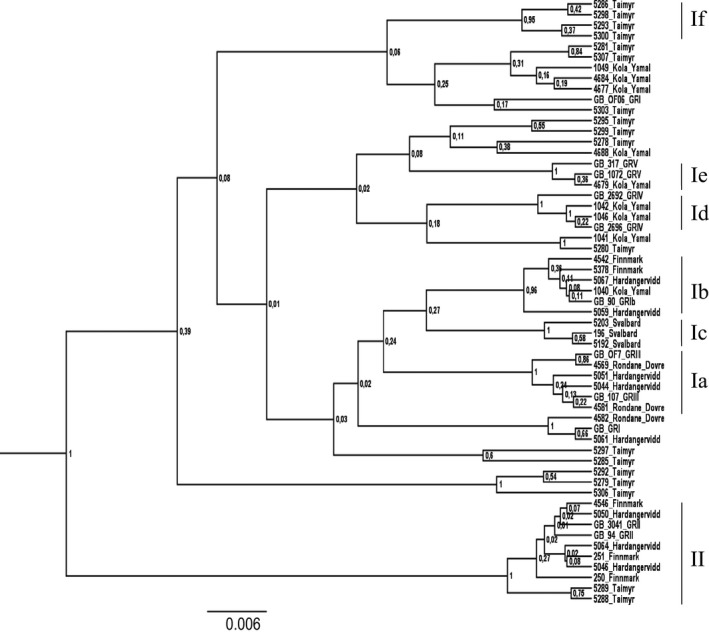
Bayesian phylogeny of the 46 CR haplotypes and subclusters **Ia**–**If** and cluster **II**. To assign haplotypes from the current study to previously described haplotype clusters, 11 haplotypes, downloaded from GenBank, were included in the analysis. A main division between clusters **I** and **II**, and six subclusters within cluster **I** was found (posterior probability values shown at each node). Subclusters **Ic** and **If** are not previously described as separate subclusters. A suggestion for renaming of CR clusters and subclusters is presented in the Appendix, due to inconsistent labeling in previous publications (Fig. A3).

**Figure A1:**
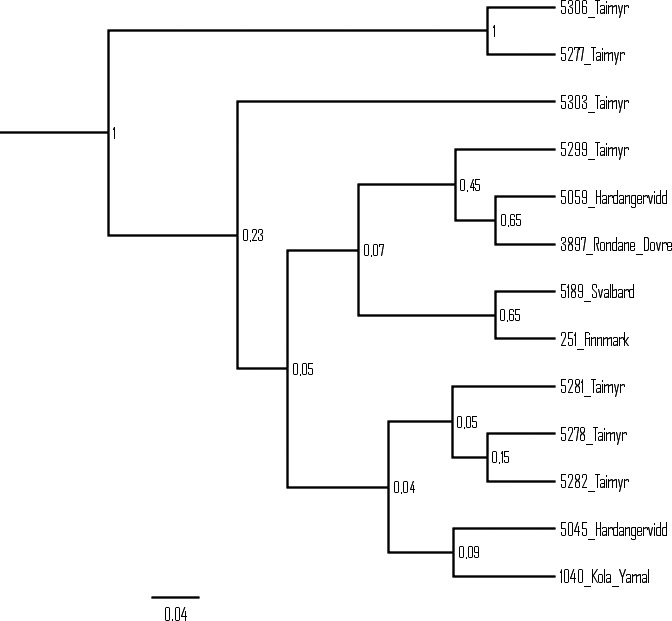
Bayesian phylogeny of the 13 cytb haplotypes (posterior probability values shown at each node). None of the clusters/subclusters identified based on the merged and CR dataset were found in the cytb phylogeny.

**Figure A2:**
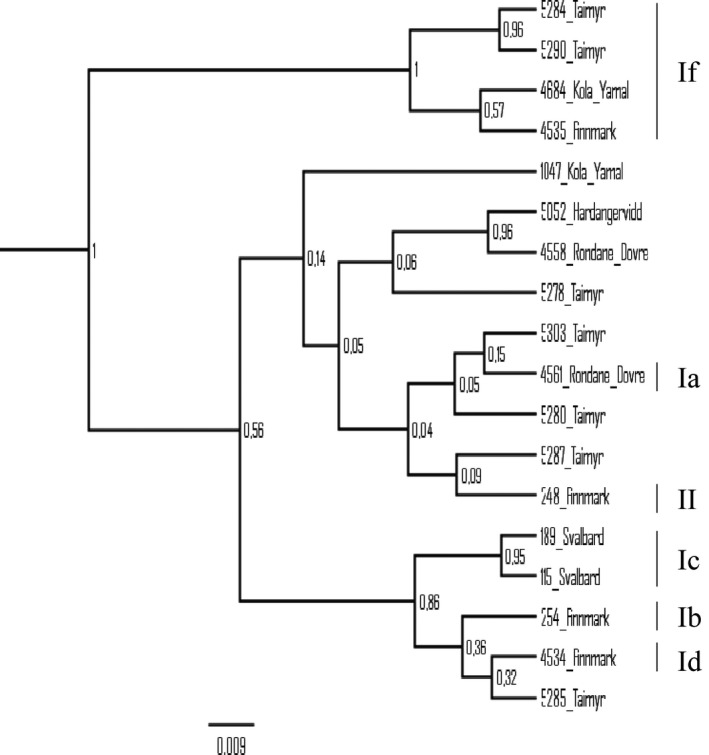
Bayesian phylogeny of the 18 COI haplotypes (posterior probability values shown at each node). Subclusters **Ia** – **Id**,** If**, and cluster **II** are identified. Support is obtained for subclusters **Ic** and **If.**

We included 11 haplotypes downloaded from GenBank to the single marker analyses of the CR, in order to identify previously described clusters. The Bayesian phylogeny generated from the 57 CR haplotypes shows several well‐defined clusters (Fig. 3). Subclusters **Ia–If** and cluster **II** obtain high support (posterior probability ≥ 0.96) and are congruent with the results from the Bayesian phylogeny based on the merged dataset and from previous studies of the CR (Figs. 2A, 3). Subcluster **If**, based on the CR, has structure similar to **If** derived from the merged dataset; however, only four of the CR haplotypes obtain support (Figs. 2A, 3). The Bayesian phylogeny of the 13 cytb haplotypes shows low resolution and none of the clusters/subcluster found in the merged and the CR phylogenies are identified (Fig. A1). The COI dataset, comprising 18 haplotypes, shows higher resolution and subclusters **Ia**,** Ib**,** Ic**,** Id**,** If** and cluster **II** are identified. Subclusters **Ia**,** Ib**,** Id** and **II** are identified as having only one haplotype. High support (≥95) is obtained for subclusters **Ic** and **If** (Fig. A2). Subcluster **If** includes the same samples (except from one individual from Finnmark, Norway) as subcluster **If** based on the merged dataset. Calculations of tMRCA for the CR subclusters **Ia**–**If** and cluster **II** gave estimates ranging from 4008 to 8603 years before present (YBP). The oldest dates are estimated for cluster **II** (8603 YBP, HPD interval: 2899–15,000) and for subcluster **If** (7848 YBP, HPD interval: 1332–16,300). The youngest dates are estimated for subcluster **Ie** (4008 YBP, HPD interval: 5170–8764) and for subcluster **Ic** (4823 YBP, HPD interval: 497–10,600) (Table 3).

**Table 3:** Mean and median tMRCA and 95% HPD interval for the different subclusters and cluster **II.**

Haplotype clusters	tMRCA YBP (mean)	tMRCA YBP (median)	95% HPD interval
Ia	6042	5554	1753–11,400
Ib	7058	6425	2065–13,400
Ic	4823	4134	497–10,600
Id	5542	4950	48–11,400
Ie	4008	3490	517–8764
If	7848	6577	1332–16,300
II	8603	7958	2899–15,000

## Discussion

### Haplotype structure as inferred from the merged dataset

The merged dataset indicates a main division between the highly diverse cluster **I**, comprising six subclusters (**Ia**–**If**), and the well‐supported cluster **II.** Cluster **Ia** comprises the ancient, wild and native haplotypes from Rondane/Dovre, which are still the dominating types in these populations (Røed et al. 2014). Subclusters **Id** and **Ie** both show only two haplotypes in the merged dataset. These are subclusters previously described from analyses of the CR and are dominated by haplotypes commonly found in domesticated Russian reindeer (Røed et al. 2008). The four haplotypes in subcluster **Ic** are unique for the Svalbard archipelago and have not previously been described to constitute a separate subcluster. Svalbard reindeer is an isolated and sedentary population of approximately 9000–11,000 animals (Øritsland 1986), showing low degree of genetic variation (hd = 0.501, *π *= 0.001 in the CR). Reduced levels of genetic variation in the Svalbard population are also reported from studies based on other markers like transferrin (Soldal and Staaland 1980; Røed 1985) and microsatellites (Côté et al. 2002; Yannic et al. 2014). In the present study, we obtained support for the Svalbard haplotypes to comprise a separate subcluster, suggesting that Svalbard reindeer have been isolated from other Beringian populations for a substantial amount of time. This finding is also supported by the tMRCA mean estimate, which indicates isolation of the Svalbard population 4823 YBP (HPD interval: 497–10,600 YBP). Haplotypes in subcluster **Ib** are in combination with haplotypes from cluster **II**, the most commonly found haplotypes in Scandinavian domestic reindeer. Subcluster **Ib** has also previously been suggested to split off from the other Beringian subclusters and to constitute a separate group (Bjørnstad et al. 2012). This finding is supported by the Bayesian phylogeny in the current study.

The merged dataset shows that the Russian haplotypes are highly diverse and appear in all clusters except **Ia** (south‐central Norway) and **Ic** (Svalbard), as well as being dominating in clusters **Ie** and **If** (Russia). The Taimyr samples seem to be especially variable (hd = 0.972, *π *= 0.017 in the CR) as shown by others (Kholodova et al. 2011; Baranova et al. 2012). This may be explained by the size of the Taimyr population, which is the largest extant Eurasian wild reindeer population, constituting 700 000–750 000 animals (Kholodova et al. 2011). The Taimyr population is probably also a historically united population which has maintained a high effective population size over a long period of time (Kholodova et al. 2011). Our results point in the direction of a separate Russian subcluster (**If**). However, due to the size and complexity of the Russian populations, more extensive sampling should be conducted before we draw conclusions regarding genetic structure within Russian reindeer.

### Comparing the results from the merged dataset and the three mitochondrial markers analyzed separately

Comparing the results derived from the merged dataset with the results from the three markers analyzed separately showed similar maternal clustering in the phylogenies, except in the phylogeny inferred from the cytb fragment. None of the subclusters found in the merged dataset could be identified by analyzing the cytb region. Low levels of genetic variation could possibly be explained by selection and the replacement of preexisting variation by selective sweeps (Avise 2000; Foote et al. 2011). However, a negative and nonsignificant Tajima's *D* estimate indicates that positive selection is an unlikely source for the low genetic variation found in this marker. Low levels of variation in this marker can probably be explained by high levels of functional constrains reflecting the central role of cytb in energy production in the mitochondria (da Fonseca et al. 2008). A longer fragment from the cytb region could have been sequenced and analyzed (see Cronin et al. 2006; Yannic et al. 2014). However, our results are congruent with the results from a study performed by Yannic et al. 2014; showing little differentiation in the cytb region within Eurasian reindeer. More structure is found when analyzing the COI region, but with low support for some of the clusters. Nevertheless, this implies that the COI region might be a more appropriate addition to the CR than the cytb region. The merged data as well as the CR dataset show high degree of structure within Eurasian reindeer. Congruence between the two phylogenies may be explained by the difference in substitution rates between the CR and the two protein‐coding markers, making the CR the most influential contributor of genetic variation. While the evolutionary rate for the cytb region is calculated to be 2.23% /MY (caribou and reindeer, Yannic et al. 2014) and 7.6% /MY for the COI region (reindeer), a substitution rate as high as 72.46% /MY has been calculated for the CR (reindeer, Røed et al. 2014). The high substitution rate in the CR is also consistent with the results from a study on the bovine control region where a substitution rate of 58.9% /MY is proposed (Ho et al. 2008).

The most recent common ancestor (MRCA) estimates obtained from the CR subclusters were all within a relatively short time period (4008 ‐ 8603 YBP). This implies that the subclusters under study diversified after the last glacial period and hence that postglacial colonization routes and isolation events have had a great impact on present‐day maternal haplotype structure. The relatively young colonization history of Eurasian reindeer makes it necessary to use highly variable markers (e.g., the CR), as the variation in more conserved markers probably reflects events from a more distant past, rather than postglacial diversification of haplotypes (Hewitt 2000). However, the high substitution rate, which makes the CR a popular marker for intraspecific phylogenetic studies, may also lead to ambiguous phylogenetic patterns and the misinterpretation of the data. Reduced resolution and genetic structure due to homoplasy have shown to be a possible methodological problem when using highly variable markers (Vandewoestijne et al. 2004; Bulgarella et al. 2010; Bradman et al. 2011). In the present study, high variability and well‐supported subclusters were found in the CR phylogeny. Several of the subclusters were also identified in the COI phylogeny, indicating that the CR has an appropriate amount of variation for studying Eurasian reindeer haplotype structure. However, subcluster **If** (Russia) showed lower resolution in the CR compared to the COI phylogeny and the phylogeny based on the merged dataset. The incongruence found between the two markers leaves subcluster **If** unresolved, and we cannot rule out the possibility of there being some degree of homoplasy within the CR dataset. As such, including slower‐evolving markers may be a useful method to reveal phylogenetic inconsistencies, even when the data are characterized by a main pattern of recent divergence.

## Concluding remarks

Bayesian phylogeny and a MJ network derived from the merged data revealed high degree of structure within Eurasian reindeer and nine clusters/subclusters were identified. We found Svalbard reindeer to constitute a separate subcluster (denoted **Ic**), implying that Svalbard reindeer have been isolated from the large continuous Beringian population for a substantial amount of time. We also found a complex structure within the Russian samples, implying that more extensive sampling is needed in order to obtain a better understanding of haplotype structure within Russian reindeer. Comparing results from the merged dataset with the results from analyses of the three markers separately shows similar clustering in all phylogenies except in the cytb phylogeny. Analyses based on the merged dataset showed topology and clustering, congruent with the results from analyzing the CR separately. However, reduced resolution was found in one of the CR subclusters (**If)** compared to the result based on the merged dataset, demonstrating the value of adding slower‐evolving loci, when dealing with highly variable markers. Nonetheless, our results imply that the CR is an appropriate marker for studying intraspecific genetic structure in Eurasian reindeer and possibly for the identification of significant phylogenetic or population units in need of separate management considerations.

## Conflict of Interest

None declared.

## Figures and Tables

**Figure A3:**
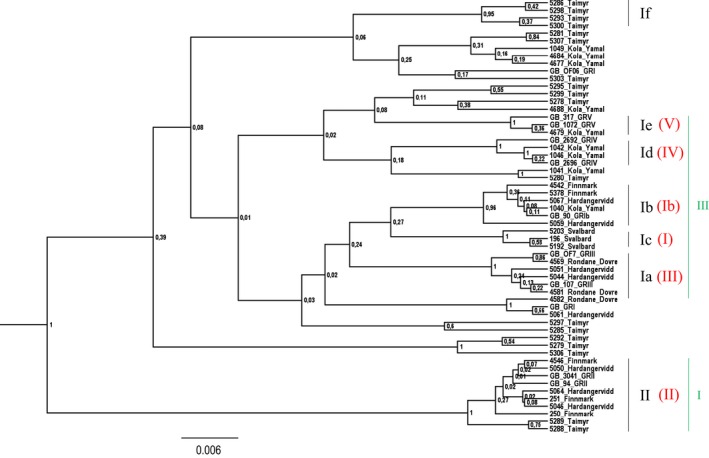
Bayesian phylogeny of the 46 CR haplotypes, in addition to 11 haplotypes representing previously described haplotypes (downloaded from GenBank). Subclusters **Ia**–**If** and cluster **II** are identified. This figure gives an overview of how the clusters/subclusters have been labeled in previous studies and in the current study. Flagstad and Røed (2003) described three main haplotype clusters, **I**–**III**, including all extant subspecies. Clusters **I** and **III** (shown in green) are further discussed in the current study. Cluster **I**, with a suggested origin from southern Europe (Flagstad and Røed 2003), has in later publications and in the current study been denoted as cluster **II** (Røed et al. 2008; Bjørnstad et al. 2012). Cluster **III** is the large variable Beringian cluster, which has been divided into five subclusters. These subclusters have in previous publications been denoted **I**,** Ib**,** III**,** IV,** and **V** (shown in red, in brackets) (Røed et al. 2008; Bjørnstad et al. 2012). Due to the inconsistent labeling of clusters and subclusters, we suggest that all haplotypes with a Beringian origin should be placed in cluster **I**. Subclusters within cluster **I** are further labeled as **Ia**–**If**, as shown in the figure (in black).
